# Differential vasoactive effects of sildenafil and tadalafil on cerebral arteries –relevant to migraine?

**DOI:** 10.1186/1471-2210-11-S1-P42

**Published:** 2011-08-01

**Authors:** Christina Kruuse, Saurabh Gupta, Elisabeth Nilsson, Lars Kruse, Lars Edvinsson

**Affiliations:** 1 Department of Clinical Experimental Research, Glostrup Hospital, Univ. Copenhagen, Denmark; 2 Department of Neurology, Glostrup Hospital, Univ. Copenhagen, Denmark; 3 Lundbeck Foundation Center for Neurovascular Signalling (LUCENS), Glostrup Hospital, Univ. Copenhagen, Denmark; 4 Division of Experimental Vascular Research, Department of Internal Medicine, University Hospital of Lund, Sweden

## Background

Phosphodiesterase 5 (PDE5) is associated with migraine pathophysiology, stroke recovery and vasospasm treatment [1,2]. We have shown previously that vasodilatation was not a prerequisite for migraine induction; sildenafil elicited migraine-like attacks in migraine patients without measurable changes in intra- or extra-cerebral artery diameter. Further, sildenafil was found to not affect neurovascular response or excitability [3]. However, dural artery responses were not accounted for in the human studies and minor vascular changes of functional importance may not have been detected.

The potential vascular interplay of PDE5 inhibitors sildenafil, tadalafil and UK-114,542 were studied by intra- versus extra-luminal administration in rat middle cerebral arteries (MCA) *in vitro* and on middle meningeal arteries (MMA) *in vivo*.

## Aim

To examine a possible vascular site of action, if any, of each of sildenafil and tadalafil by investigating 1) the effects of PDE5 inhibitors in vitro dilatation of the middle cerebral artery (MCA) with controlled luminal or extra-luminal application of the drugs and 2) the in vivo effects of intravenous PDE5 inhibitors on the middle meningeal artery (MMA) dilatation in a closed cranial window model in rats.

## Methods

Rat MCA diameter was investigated using pressurised arteriography, applying UK-114,542, sildenafil, and tadalafil intra- or extra-luminally. Effects on MMA were studied in the *in vivo* closed cranial window model.

## Results

At high concentrations, abluminal sildenafil and UK-114,542, but not tadalafil, induced dilatation. Luminal application elicited a contraction of 4 % (sildenafil, p = 0.03) and 10 % (tadalafil, p = 0.02). *In vivo*, sildenafil, but not tadalafil, dose-dependently dilated MMA concomitant to blood pressure reduction (1-3 mg/kg);1 mg/kg sildenafil inducing 60 ± 14 % (p = 0.04) and vehicle (DMSO) 13 ± 6 % dilatation.

## Conclusion

PDE5 inhibitors applied luminally had contractile effect on MCA. Abluminal sildenafil induced MCA dilatation above therapeutic levels. *In vivo*, sildenafil dilated MMA. Tadalafil had no dilatory effects. PDE5 inhibitors show differential vascular activity in arteries, although clinically the potential for headache induction appears similar. Such findings support clinical studies showing no vasodilatory effects of sildenafil on cerebral arteries in healthy subjects.
